# Persistent Increase in Serum Alkaline Phosphatase in a Patient with Monoclonal Gammopathy of Undefined Significance

**DOI:** 10.1155/2020/8406971

**Published:** 2020-01-31

**Authors:** I. Ramasamy

**Affiliations:** Worcester Royal Hospital, Charles Hastings Way, Worcester WR41DD, UK

## Abstract

We report the finding of alkaline phosphatase-immunoglobulin complex (macro-alkaline phosphatase (macro-ALP)) in a patient with persistently increased ALP activity. The identification of macro-ALP is important to rule out pathological causes of increased ALP activity and to avoid unnecessary diagnostic investigation. The patient was subsequently diagnosed with vitamin D deficiency, gallstone pancreatitis, and monoclonal gammopathy of undefined significance (MGUS). Macro-ALP can coexist with disease that can increase serum ALP activity. We report, for the first time, a case of macro-ALP in a patient with a monoclonal protein (M-protein).

## 1. Introduction

The tissue-nonspecific alkaline phosphatase (ALP) is the major fraction found in the serum and is coded by a single gene expressed in the liver, bone, and kidney. Intestinal alkaline phosphatase, which is about 25% of normal sera, is coded by a separate gene which is separate from the gene that codes for placental alkaline phosphatase and the placental-like isoenzyme (which is an oncofetal form the enzyme). Raised serum ALP may be due to the liver ALP isoenzyme as a result of hepatic damage or cholestatasis. Increased bone-specific ALP can be caused by a number of pathologies affecting the bone: fracture, Paget's disease, osteomalacia, and bone metastases. Raised intestinal ALP has been reported in liver cirrhosis, diabetes, chronic kidney disease, and bowel ischemia and infarction. Alkaline phosphatase isoenzyme analysis is used to understand the changes in serum values of ALP and has many diagnostic advantages in identifying underlying pathology [1, 2].

Rarely increased serum ALP activity will be due to an immunoglobulin-bound form of ALP (macro-ALP). Macro-ALP has reduced renal clearance usually due to a high molecular weight form of the enzyme. The importance for the diagnosis of macro-ALP lies in their ability to cause diagnostic confusion when plasma ALP activities are elevated, leading to unnecessary and potentially harmful investigations [3]. We report here, to our knowledge for the first time, the finding of macro-ALP in a patient with monoclonal gammopathy of undefined significance (MGUS).

## 2. Case Presentation

**Figure 1:**
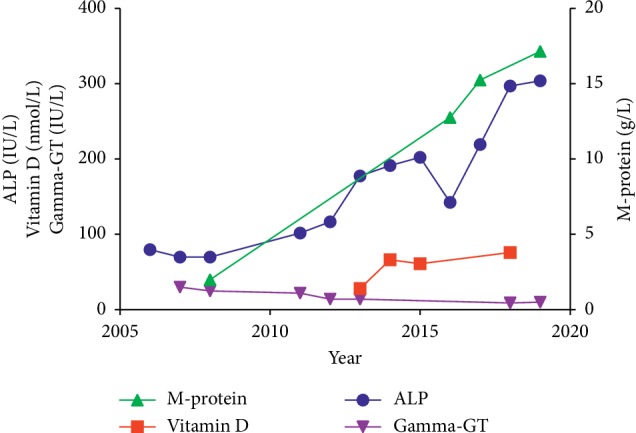
Changes in ALP, M-protein, vitamin D, and gamma-GT in the patient's sera with time.

A 73-year-old man with a total ALP of 70 IU/L (reference range (RR) 30–130, using p-nitrophenyl phosphate as a substrate, Roche UK) was found to have a minor IgG kappa monoclonal band (M-band) of 2.8 g/L in 2009. His total ALP increased to 142 IU/Lin 2012 and to 178 IU/L in 2013 (Figure 1). His gamma-glutamyl transferase (gamma-GT, RR 10–70 IU/L), alanine aminotransaminase, and total bilirubin remained within the reference range. His 25-hydroxy vitamin D level was found to be 28.2 nmol/L (RR > 50 nmol/L for adequate vitamin D status). He was treated with vitamin D. Vitamin D status was assessed as adequate in Feb 2014 at 66.5 nmol/L. His 25-hydroxy vitamin D concentration remained at 76.2 nmol/L in 2018, with a parathyroid hormone level of 29 ng/L (RR 15–65), and his unadjusted calcium was 2.3 mmol/L (RR 2.2–2.6). His full blood count profile and renal function remained within the reference limit. The ALP remained persistently high at 192 IU/L and remained high during 2015 at 174 IU/L. In March 2016, his M-band was 15.3 g/L, kappa light chain was 56.6 mg/L (RR 3.3–19.4 mg/L), lambda light chain was 31.1 mg/L (RR 5.7–26.3 mg/L), and the ratio was 1.821 (RR 0.26–1.65). Progression in his M-protein is shown in Figure 1. He presented with acute pancreatitis in 2017. Radiological investigation identified multiple gallstones within the gallbladder leading to a diagnosis of gallstone pancreatitis. He was admitted for laparoscopic cholecystectomy and remained well following surgery. He had an extensive past medical history which included hypertension, chronic obstructive pulmonary disease, diabetes, and total knee replacement. His treatment included amlodipine, vitamin D, clopidogrel, omeprazole, pravastatin, and lisinopril.

Following cholecystectomy, his ALP remained raised, and alanine aminotransaminase, bilirubin, gamma-GT, serum calcium, and renal function were within the reference limit. Liver screening for antinuclear antibody, mitochondrial antibody, smooth muscle antibody, and anti-liver-kidney microsomal antibody was negative. Referral to a hospital gastroenterologist and an abdominal ultrasound detected a post inflammatory pancreatic cyst but did not report liver disease.

## 3. Investigations

**Figure 2:**
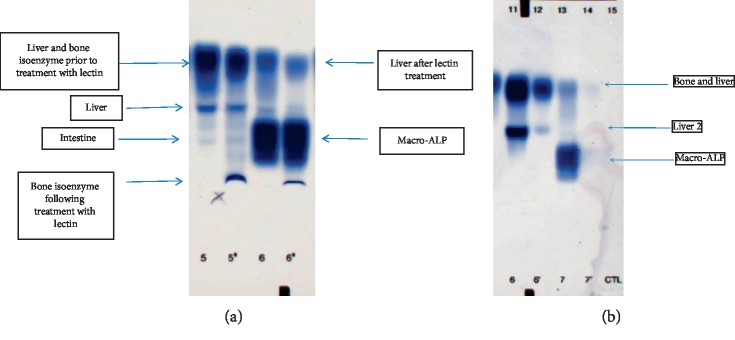
(a) ALP isoenzyme analysis following lectin treatment. Lane 5: Control sera with liver, bone, and intestinal isoenzyme. Lane 5′: control sera following lectin treatment. Lane 6: the patient's sera. Lane 6′: the patient's sera following treatment with lectin. Lectin precipitates the bone isoenzyme, allowing the liver isoenzyme to be quantitated. Electrophoretic mobility of the macro-ALP is not affected by lectin. (b) ALP isoenzyme analysis following PEG precipitation. Lane 6: control with liver ALP isoenzyme. Lane 6′: control following treatment with PEG. Lane 7: the patient's sera. Lane 7′: the patient's sera following treatment with PEG. PEG treatment precipitates the macroenzyme in the patient's sera.

ALP isoenzyme analysis was carried out by the Sebia (UK) serum enzyme electrophoresis method. The SEBIA system utilizes the different degrees of sialylation of liver and bone isoenzymes to separate them. Wheat germ lectin presents a strong affinity for the sialic acid residues and consequently binds preferentially to the bone isoenzyme that is sialated to a higher degree. The separated ALP isoenzymes are visualized at 37°C and 50°C using an ALP-specific chromogenic substrate, 5-bromo-4-chloro-3-indolyl phosphate/nitro blue tetrazolium in aminomethyl propanol (AMP) buffer, with pH 10.1. Isoenzyme analysis of the patient's sera in 2018 identified an unusual form of ALP (Figure 2(a)).

Further investigation showed that the electrophoretic mobility of the unusual alkaline phosphatase isoform was not affected by lectin. Equal volumes of the patient's sera and poly ethylene glycol (PEG) 6000 (240 g/L in 9 g/L of saline) were mixed, and the residual activity was measured. Fifteen patient sera with liver, bone, and intestinal alkaline phosphatase isoenzymes were used as controls. The average measured residual activity was 95.4% in controls (range 84.8–108.5), compared to 25.2% (*n* = 3) in the patient. Isoenzyme electrophoresis of the patient's sera before and after PEG precipitation showed that the unusual form was precipitated by PEG and identified an immunoglobulin-alkaline phosphatase macroenzyme complex (Figure 2(b)).

**Figure 3:**
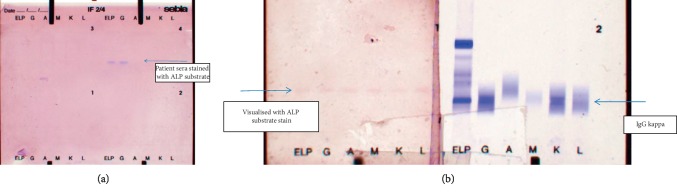
(a) Immunofixation gels stained with alkaline phosphatase substrate. Three control sera and the patient's sera were electrophoresed using the Sebia immunofixation gel and stained with ALP substrate. Gel 1–3: three control sera on all lanes. Gel 4: lanes 1 and 2: the patient's sera. (b) Comparative stain of the immunofixation gel with alkaline phosphatase substrate and anti-IgG/A/M heavy chain and kappa/lambda light chain. Gel 1: lanes 1–6: the patient's sera stained with alkaline phosphatase substrate. Gel 2: lanes 1–6: the patient's sera stained with antisera to immunoglobulin heavy chain and light chains.

The Sebia (UK) immunofixation method allows the separation and characterization of M-protein heavy and light chains using specific antisera. Following agarose gel electrophoresis, the gel is incubated with antisera to IgG, IgA, and IgM heavy chains and kappa and lambda light chains. The excess antisera are removed, the precipitated (immunofixed) immunoglobulin is visualised, and the M-protein is identified by staining. The patient's sera and three controls with total alkaline phosphatase in the range 200–300 IU/L were electrophoresed on the immunofixation gel (Sebia, UK). Following electrophoresis serum, ALP was visualised using the specific substrate for ALP. Visualisation was performed by a manual technique in which the gel was overlaid with the substrate and incubated at 37°C. A discrete band that was visualised by the ALP chromogenic substrate was identified in the patient's sera but not in controls (Figure 3(a)). Comparison with the gel visualised with antisera to immunoglobulin heavy and light chains showed that the band had a slightly higher mobility than the IgG kappa M-band found in the patient's sera (Figure 3(b)).

The discrete band found using specific substrate staining following electrophoresis on the immunofixation gel suggested a monoclonal (M) component to the ALP-immunoglobulin complex. The molecular weight of IgG is 150,000 and of the non-tissue-specific form of ALP is 186,000 [4]. It is likely that the electrophoretic mobility of the immunoglobulin-ALP complex differs from the IgG M-protein. It is probable at the activity of 180 IU/L, and the concentration of the macro-ALP in mg/L is below the detection limit of the immunofixation technique (manufacturer detection limit: 150 mg/L by precipitation using the anti-IgG sera) [5].

## 4. Discussion

There have been several reports of macro-ALP in the literature. Reported macro-ALP were bound to IgG [6, 7] and to a lesser extent IgA [8, 9]. The ALP is usually of liver and bone origin or both [10]. Gel filtration analysis of the macro-ALP suggested that the results will be consistent with equimolar complexes of IgG-ALP or a complex made up of two molecules of IgG with one of ALP or one molecule of IgG and two molecules of ALP [6]. Wenham et al. [9] reported two peaks on gel chromatography and suggested different molar ratios of ALP and IgA immunoglobulin complexes as well as lipid forms within the macro-ALP complex. Maekawa et al. [8] suggested that ALP was attached through the F (ab′) 2 fragment of IgA and that the molecular weight of the complexes suggested that 2 molecules of ALP was associated with one molecule of divalent IgA.

Characterisation of the macro-ALP was performed by gel filtration [7], immunoelectrophoresis [7], PEG precipitation [11], and proteolytic digestion [9]. Protein A/G agarose affinity chromatography was used to demonstrate the presence of macro-ALP [12]. This method will not detect complexes to immunoglobulins other than IgG so has limitations [10]. In a further case study, Nagakawa et al. [13] reported an intestinal ALP-IgG complex in which the IgG had the ability to inhibit ALP in a patient's serum. The electrophoretic mobility of the unusual form of ALP found in this patient coincided with that of the intestinal enzyme. However, we confirmed the presence of the immunoglobulin-ALP complex by PEG precipitation. The presence of a discrete band detected by specific ALP substrate stain in the gamma region following protein electrophoresis was further supportive of an immunoglobulin-ALP complex.

Macro-ALP has been reported in patients with ulcerative colitis [11], carcinoma of prostate [10], bronchopneumonia and recurrent infections [9], and liver cirrhosis and cholestasis [6]. Atypical ALP with posttranslational modification has been described in a patient with Hodgkin's disease [14]. There was no observed correlation with the presence of the macro-ALP and clinical abnormalities. The question as to why autoantibodies to ALP occur in sera is not fully understood at present.

Isolated increase in ALP is associated with a variety of medical illnesses. In the first instance, a reasonable approach would be a careful medical and drug history and physical examination. The within-subject variation for ALP was reported as 6.4% [15], suggesting minor increases in ALP can be within biological variation. Raised ALP of unknown cause requires further tests to define associated pathology.

The finding of an isolated increase in ALP triggered several investigations in this patient. The increase was at first attributed to vitamin D deficiency. The persistent increase following repletion with vitamin D resulted in carrying out further investigations for liver disease and a referral to a hospital based specialist gastroenterologist. Conditions that favour the presence of a macro-ALP are the absence of symptoms, an isolated and persistently increased ALP, symptoms atypical for the abnormal level of the enzyme. However, as in this patient, the presence of macro-ALP can coexist with disease that can cause an increase in ALP, vitamin D deficiency, gallstone pancreatitis, and MGUS. Identification of macro-ALP in this context becomes of relevance in the correct interpretation of increased total ALP. Interestingly, macro-ALP in this patient raises the possibility of ALP bound to the M-protein as a cause of the immunoglobulin-ALP complex.

Recent studies have indicated an increased fracture risk in multiple myeloma around the time of presentation [16]. MGUS usually precedes multiple myeloma, and the risk of fractures is increased in MGUS patients [17]. The consequences of skeletal involvement are severe pain, spinal cord compressions, and bone fractures which have a dramatic impact on a patient's quality of life. In serial monitoring of patients with skeletal metastases bone, ALP correlated with progression or regression of metastatic spread [18]. In multiple myeloma, the bone remodelling balance is disrupted and osteolytic lesions develop. In multiple myeloma, the effects of upregulated osteoclastogenesis is amplified by the inhibition of osteoblast activity [19]. Changes in bone remodelling may be monitored by the detection of specific serum markers, including carboxy-terminal telopeptide of type 1 collagen, *β*-crosslaps, and deoxypyridinoline characteristic of bone degradation as well as bone-specific alkaline phosphatase, osteocalcin, amino-terminal propeptide of type I collagen, and carboxy-terminal propeptide of type I collagen characteristic of bone formation [20]. In a goal in which state-of-the-art treatment strategies are used to restore balanced bone remodelling, biochemical markers of bone remodelling may play a role in slowing down bone damage. In this clinical scenario, identification of a macro-ALP becomes important to prevent unnecessary investigation and incorrect interpretation of increased total ALP.

In summary, we report a case of macro-ALP in a patient with IgG kappa MGUS. Diagnostic use of ALP isoenzyme characterisation is of value and can provide useful information on underlying pathology. The presence of macro-ALP can confuse the interpretation of increased total ALP and can lead to unnecessary investigation. This is particularly true in disease states, such as MGUS and multiple myeloma which can lead to pathology that can result in increased total ALP.

## 5. Learning Points

1. Differential diagnosis of an isolated increased ALP can be a challenge and can trigger several investigations
2. We report for the first time, macro-ALP in a patient with M-protein
3. Macro-ALP can coexist with several pathologies which can increase ALP
4. It is important to detect the presence of macro-ALP as they can cause diagnostic confusion leading to several unnecessary investigations
